# Notch, metabolism and macrophages

**DOI:** 10.18632/oncotarget.4651

**Published:** 2015-06-25

**Authors:** Jun Xu, Feng Chi, Hidekazu Tsukamoto

**Affiliations:** Southern California Research Center for ALPD and Cirrhosis, and Department of Pathology, The Keck School of Medicine of The University of Southern California, Los Angeles, CA, USA

**Keywords:** Immunology, notch, macrophage, metabolism, inflammation, Immunology and Microbiology Section, Immune response, Immunity

Notch signaling is a highly conserved and critical pathway that integrates environmental cues to specify cell fate during development. Notch is a single transmembrane receptor with 4 isoforms (Notch1 to 4) and five known Notch ligands. Notch activation is initiated by ligand binding, which leads to sequential proteolytic cleavage and liberation of Notch intracellular domain (NICD) from the membrane. The NICD translocates into the nucleus where it interacts with a DNA binding protein CSL and other nuclear proteins to form a transcriptional co-activator complex [1]. Recent studies have shown that Notch pathway is also involved in liver regeneration/repair, carcinogenesis, metabolism, and modulation of inflammatory response by regulating macrophage polarization through toll-like receptor 4 and/or NF-κB pathway [2]. It is now clear that Notch functions in cell fate determination extend beyond development.

Emerging evidence suggests that metabolic reprogramming severs as intrinsic signal to regulate cell differentiation and proliferation [3]. This concept has been demonstrated in macrophages undergoing differentiation. Metabolic reprograming to glycolysis is observed in macrophages polarized to proinflammatory (M1) phenotype as compared to fatty acid oxidation in alternatively activated (M2) macrophages [4]. Glycolysis converts glucose to pyruvate, which is further metabolized in cytoplasm under hypoxia to produce lactate. In the presence of oxygen sufficiency, pyruvate is converted in mitochondria to acetyl-CoA, which enters tricarboxylic acid (TCA) cycle to generate reducing equivalent nicotinamide adenine dinucleotide (NADH) for oxidative phosphorylation (OXPHOS). Metabolic switch to glycolysis, however, is energetically unfavorable for energy consuming phagocytosis of M1 macrophages, because glucose metabolism through OXPHOS generate ~18 fold more ATP than through glycolysis. In fact, macrophages are known to rely a significant degree on OXPHOS during phagocytosis and in response to *Listeria monocytogenes* challenging [5]. Thus, we have investigated the metabolic phenotype in M1 macrophages and whether metabolic reprograming is causally linked to M1 macrophage activation.

Our recent study, published in the Journal of Clinical Investigation [6], has confirmed that M1 activation of hepatic macrophages *in vivo* is Notch1 dependent using a mouse model of alcoholic steatohepatitis. We show that NICD1 translocate to both nucleus and mitochondrion in LPS/IFNγ-stimulated M1 macrophages. In nucleus, NICD1 binds to promoter region of M1 genes such as Nos2, Tnf, and Il-17rc, and directly activates transcription of these genes. In mitochondria, NICD1 is enriched in the promoter region of mtDNA displacement loop (D-loop), and expression of mtDNA-encoded electronic transport chain (ETC) components (NADH dehydrogenase, cytochrome b, cytochrome c oxidase, and ATP synthases) is upregulated in M1 macrophages in a manner dependent on Notch1 activation. These cells have increased oxygen consumption rate and mtROS generation, suggesting increased OXPHOS activity. As expected, M1 macrophages have increased glucose uptake and lactate production, but both of which are prevented by Notch inhibition, indicating Notch-dependent metabolic switch to aerobic glycolysis. Interestingly, these cells have a concomitant ~23% increase in glucose flux to TCA cycle, which would produce 4.2-fold more ATP than that of glycolysis and provide NADH for ECT and OXPHOS. We further demonstrate that Notch1 directly upregulates expression of pyruvate dehydrogenase (PDH) phosphatase catalytic subunit 1 (PDP1), which dephosphorylates and activates PDH to enhance glucose metabolism through pyruvate to TCA cycle for OXPHOS. More importantly, Notch1 KO, glycolytic inhibitor 2-deoxyglucose, or *Pdp1* shRNA-silencing reduces mtROS and M1 gene expressions. These results establish a Notch1 dependent metabolic axis of glucose-pyruvate-OXPHOS and subsequent generation of mtROS, which links Notch dependent glucose metabolism to M1 macrophage activation [6].

We conclude that Notch1 pathway regulates macrophage M1 fate through two mechanisms: 1) Notch1 upregulates M1 genes through transcription activation; and 2) Notch1 reprograms macrophage metabolism to glucose OXPHOS and subsequent generation of mtROS, which enhances M1 gene expressions. More importantly, the observation of Notch1 dependent upregulation of Pdp1, and the decreased mtROS and M1 gene expressions by Pdp1 silencing demonstrate the causal link of metabolic reprograming to macrophage M1 fate. In addition, our unpublished results and studies by other groups have shown glutamine metabolism through TCA cycle is also critical for M1 macrophage activation. Interestingly, glycolytic inhibitor 2-deoxyglucose can prevent the glutamine dependent M1 macrophage activation [7]. Thus, further studies detailing the role of Notch1 in regulation of different TCA anaplerotic fluxes will provide new insight into the mechanisms of metabolic determination of cell fate.
